# Fixed point results for fractal generation in Noor orbit and s-convexity

**DOI:** 10.1186/s40064-016-3530-5

**Published:** 2016-10-21

**Authors:** Sun Young Cho, Abdul Aziz Shahid, Waqas Nazeer, Shin Min Kang

**Affiliations:** 1 Department of Mathematics and RINS, Gyeongsang National University, Jinju, 52828 Korea; 2 Department of Mathematics and Statistics, University of Lahore, Lahore, Pakistan; 3 Division of Science and Technology, University of Education, Lahore, Pakistan

**Keywords:** Noor orbit, Julia set, Mandelbrot set, S-convexity, 47H10

## Abstract

In this note, we give fixed point results in fractal generation (Julia sets and Mandelbrot sets) by using Noor iteration scheme with s-convexity. Researchers have already presented fixed point results in Mann and Ishikawa orbits that are examples of one-step and two-step feedback processes respectively. In this paper we present fixed point results in Noor orbit, which is a three-step iterative procedure.

## Background

In 1918, Gaston Julia investigated the iteration process of complex function and attained a Julia set. Julia sets are striking examples of computational experiments that were far ahead of its time. These mathematical objects were seen when computer graphics became available (Peitgen et al. 2004). In 1979, Benoit Mandelbrot introduced the Mandelbrot set by using the complex function $z^{2}+c$ with using *c* as a complex parameter and *z* as a complex function (Mandelbrot 1982). The visual beauty, complexity and self similarity of these objects have made a field of intense research nowadays. Convexity and its generalization plays a vital role in different parts of mathematics, mainly in optimization theory. Presented paper deal with approximate convexity, a common generalization of s-convexity and results of Bernstein and Doetsch (1915). The concept of s-convexity and rational s-convexity was introduced by Breckner and Orb’an (1978). In 1978, Breckner and Orb’an (1978), Hudzik and Maligranda (1994) proved that when $0<s<1,$ s-convex functions are nonnegative moreover as *s* decreases the set of s-convex functions increases.

In 1994, Hudzik and Maligranda (1994) explained a few results connecting with s-convex functions in second sense. Some new results for s-convex functions about Hadamard’s inequality are discussed in Alomari and Darus (2008a, b), Kirmaci (2007). Takahashi (1970) introduced a notion of convex metric space and in 1999, for s-convex functions in second sense, (Dragomir and Fitzpatrick 1999) proved a variant of Hermite–Hadamard’s inequality. The fractal structures of Julia and Mandelbrot sets for quadratic, cubic, and higher degree polynomials, by using Picard orbit have been demonstrated in Devaney (1992). In 2004, Rani and Kumar (2004a, b) introduced superior Julia and Mandelbrot sets using Mann iteration scheme. Rana et al. (2010a, b) introduced relative superior Julia and Mandelbrot sets using Ishikawa iteration scheme. Also, relative superior Julia sets, Mandelbrot sets and tricorn and multicorns by using S-iteration scheme are presented in Kang et al. (2015a). Recently, (Ashish and Chugh 2014) introduced Julia and Mandelbrot sets using Noor iteration scheme which is a three-step iterative procedure.

Fixed point results can be found in the generation of the different types of fractals: for example, Iterated Function Systems (Prasad and Katiyar 2011; Singh et al. 2011), V-variable fractals (Singh et al. 2011), Inversion fractals (Gdawiec 2015) and Biomorphs (Gdawiec et al. 2016). Some polynomiographs are types of fractals which can be obtained via different iterative schemes, for more detail (see Kang et al. 2015c, 2016; Rafiq et al. 2014; Kotarski et al. 2012; Nazeer et al. 2016) and references therein. Kang et al. (2015b) introduced new fixed point results for fractal generation in Jungck Noor orbit with s-convexity. Mishra et al. (2011a, b) develop fixed point results in relative superior Julia sets and tricorn and multicorns by using Ishikawa iteration with s-convexity. Nazeer et al. (2015) introduced fixed point results in the generation of Julia and Mandelbrot sets.

In this paper we present some fixed point results for Julia and Mandelbrot sets by using Noor iteration scheme with s-convexity. The results of Ashish and Chugh (2014) are a special case of the results of this paper for $s = 1,$ so in this article we extend the results from Ashish and Chugh (2014). We define the Noor orbit and escape criterions for quadratic, cubic, and $k+1$th degree polynomials by using Noor iteration with s-convexity.

## Preliminaries

### **Definition 1**

(*see* Barnsley 1993 *, Julia set*) Let $f:C\longrightarrow C$ symbolize a polynomial of degree $\ge 2$. Let $F_{f}$ be the set of points in *C* whose orbits do not converge to the point at infinity. That is, $F_{f}=\{x\in C:\{\left| f^{n}(x)\right| ,$ *n* varies from 0 to $\infty \}$ is bounded$\}.$ $F_{f}$ is called as filled Julia set of the polynomial *f*. The boundary points of $F_{f}$ can be called as the points of Julia set of the polynomial *f* or simply the Julia set.

### **Definition 2**

(*see* Devaney 1992 *, Mandelbrot set*) The Mandelbrot set *M* consists of all parameters *c* for which the filled Julia set of $Q_{c}(z)=z^{2}+c$ is connected, that is

1 $$M=\left\{ c\in C:\; F_{Q_{c}}\;\text {is connected}\right\},$$

In fact, *M* contains an enormous amount of information about the structure of Julia sets. The Mandelbrot set *M* for the quadratic $Q_{c}(z)=z^{2}+c$ is defined as the collection of all $c\in C$ for which the orbit of the point 0 is bounded, that is

2 $$M=\left\{ c\in C:\left\{ Q_{c}^{n}(0)\right\} ;\quad n=0,1,2,\ldots \text {is bounded}\right\},$$

We choose the initial point 0, as 0 is the only critical point of $Q_{c}$.

### **Definition 3**

Let *C* be a nonempty set and $f:C\rightarrow C$ . For any point $z_{0}\in C$, the Picard’s orbit is defined as the set of iterates of a point $z_{0}$, that is;

3 $$O\left( f,z_{0}\right) =\left\{ z_{n};z_{n}=f\left( z_{n-1}\right) ,\quad n=1,2,3,\ldots \right\}.$$

where the orbit $O(f,z_{0})$of *f* at the initial point $z_{0}$ is the sequence $\{f^{n}z_{0}\}$.

### **Definition 4**

(*see* Noor 2000 *, Noor orbit*). Consider a sequence $\{z_{n}\}$ of iterates for initial point $z_{0}\in C$ such that

4 $$\begin{aligned}&\{z_{n+1} :z_{n+1}=\left( 1-\alpha _{n}\right) z_{n}+\alpha _{n}fu_{n};u_{n}=\left( 1-\beta _{n}\right) z_{n}+\beta _{n}fv_{n}; \\&v_{n} =\left( 1-\gamma _{n}\right) z_{n}+\gamma _{n}fz_{n};\quad n=0,1,2,\ldots \}, \end{aligned}$$

where $\alpha _{n},\beta _{n},\gamma _{n}\in [0,1]$ and $\{\alpha _{n}\},\{\beta _{n}\},\{\gamma _{n}\}$ are sequences of positive numbers. The above sequence of iterates is called Noor orbit, which is a function of five arguments $(f,z_{0},\alpha _{n},\beta _{n},\gamma _{n})$ which can be written as $NO(f,z_{0},\alpha _{n},\beta _{n},\gamma _{n})$.

## Main results

The definition of the Mandelbrot set gives us an algorithm for computing it. We simply consider a square in the complex plane. We overlay a grid of equally spaced points in this square. Each of these points is to be considered a complex *c*-value. Then, for each such *c*, we ask the computer to check whether the corresponding orbit of 0 goes to infinity (escapes) or does not go to infinity (remains bounded). In the former case, we leave the corresponding c-value (pixel) white. In the latter case, we paint the *c*-value dark. Thus the dark points represent the Mandelbrot set. Indeed, it is not possible to determine whether certain *c*-values lie in the Mandelbrot set. We can only iterate a finite number of times to determine if a point lies in M . Certain *c*-values close to the boundary of M have orbits that escape only after a very large number of iterations.

### **Corollary 1**

(The Escape Criterion) *Suppose* |*c*| *is less than or equal to* 2. *If the orbit of* 0 *under* $z^{2}+c$ *ever lands outside of the circle of radius 2 centered at the origin, then this orbit definitely tends to infinity*.

When calculating Julia sets, *z* is a variable representing a Cartesian coordinate within the image and *c* is a constant complex number, *c* does not change during the calculation of the entire image. However, when different values of *c* are used, different images representing different Julia sets will result.

The escape criterion plays a vital role in the generation and analysis of Julia sets and Mandelbrot sets. We now define escape criterions for Julia sets and Mandelbrot sets in Noor orbit with s-convexity.

We take $z_{o}=z\in \mathbb {C}$, $\alpha _{n}=\alpha ,\beta _{n}=\beta$ and $\gamma _{n}=\gamma$ then can write Noor iteration scheme with s-convexity in the following manner where $Q_{c}(z_{n})$ be a quadratic, cubic or *n*th degree polynomial.

5 $$\begin{aligned} z_{n+1}= &\,\,(1-\alpha )^{s}z_{n}+\alpha ^{s}Q_{c}(u_{n}) \\ u_{n}= &\,\,(1-\beta )^{s}z_{n}+\beta ^{s}Q_{c}(v_{n}) \\ v_{n}= &\,\,(1-\gamma )^{s}z_{n}+\gamma ^{s}Q_{c}(z_{n}) \end{aligned}$$

where $$0<\alpha ,\beta ,\gamma \le 1$$ and $$0<s\le 1.$$

We used the notion $NO_s(Q_c, 0, \alpha , \beta ,\gamma ,s)$ for the Noor iteration with s-convexity.

### Escape criterion for quadratic polynomial

#### **Theorem 1**

*Suppose that* $|z|\ge |c|>\frac{2}{s\alpha }$, $|z|\ge |c|>\frac{2}{s\beta }$ *and* $|z|\ge |c|>\frac{2}{s\gamma }$ *where* *c* *be a complex number and* $0<\alpha ,\beta ,\gamma \le 1$. *Let* $\ u_{\circ }=u,v_{\circ }=v$ *and* $z_{\circ }=z$ *then for iteration* (5) *and* $Q_c(z) = z^2 + c$ *we have* $\left| z_{n}\right| \rightarrow \infty$ *as* *n* $\rightarrow \infty .$

#### *Proof*

Consider

$$\begin{aligned} \left| v\right| =\left| \left( 1-\gamma \right) ^{s}z+\gamma ^{s}Q_{c}(z)\right| \end{aligned}$$

For $$Q_{c}(z)=z^{2}+c,$$

$$\begin{aligned} \left| v\right|= &\, \left| \left( 1-\gamma \right) ^{s}z+\gamma ^{s}\left( z^{2}+c\right) \right| \\= &\,\,\left| \left( 1-\gamma \right) ^{s}z+\left( 1-\left( 1-\gamma \right) \right) ^{s}\left( z^{2}+c\right) \right| \end{aligned}$$

By binomial expansion upto linear terms of $\gamma$ and $(1-\gamma )$, we obtain

6 $$\begin{aligned} \left| v\right|\ge &\, \left| \left( 1-s\gamma \right) z+\left( 1-s\left( 1-\gamma \right) \right) \left( z^{2}+c\right) \right| \\\ge &\, \left| (1-s\gamma )z+(1-s+s\gamma )(z^{2}+c)\right| \\\ge &\, \left| (1-s\gamma )z+s\gamma (z^{2}+c)\right| ,\quad\text {because}\,1-s+s\gamma \ge s\gamma \\\ge &\, \left| s\gamma z^{2}+(1-s\gamma )z\right| -\left| s\gamma c\right| \\\ge &\, \left| s\gamma z^{2}+(1-s\gamma )z\right| -\left| s\gamma z\right| ,\quad\text {because}\,|z|\ge |c| \\\ge &\, \left| s\gamma z^{2}\right| -\left| (1-s\gamma )z\right| -\left| s\gamma z\right| \\= &\, \left| s\gamma z^{2}\right| -\left| z\right| +\left| s\gamma z\right| -\left| s\gamma z\right| \\\ge &\, \left| z\right| (s\gamma \left| z\right| -1). \end{aligned}$$

and

7 $$\begin{aligned} \left| u\right|= &\, \left| (1-\beta )^{s}z+\beta ^{s}Q_{c}(v)\right| \\= &\, \left| (1-\beta )^{s}z+(1-(1-\beta ))^{s}(v^{2}+c)\right| , \end{aligned}$$

By binomial expansion upto linear terms of $\beta$ and $(1-\beta ),$ we obtain

8 $$\begin{aligned} \left| u\right|\ge &\, \left| (1-s\beta )z+(1-s(1-\beta ))(v^{2}+c)\right| \\\ge &\, \left| (1-s\beta )z+(1-s+s\beta )(v^{2}+c)\right| \\\ge &\, \left| (1-s\beta )z+s\beta ((\left| z\right| (s\gamma \left| z\right| -1))^{2}+c)\right| ,\quad\text { because }1-s+s\beta \ge s\beta \end{aligned}$$

Since $|z|>2/s\gamma$ implies $s\gamma \left| z\right| -1>1$ and $\left| z\right| ^{2}(s\gamma \left| z\right| -1)^{2}>$ $\left| z\right| ^{2}$ using this in (8) we have

9 $$\begin{aligned} \left| u\right|\ge &\, \left| (1-s\beta )z+s\beta (\left| z\right| ^{2}+c)\right| \\\ge &\, \left| s\beta z^{2}+(1-s\beta )z\right| -\left| s\beta c\right| \\\ge &\,\left| s\beta z^{2}+(1-s\beta )z\right| -\left| s\beta z\right|,\quad \text { because }|z|\ge |c| \\\ge &\,\left| s\beta z^{2}\right| -\left| (1-s\beta )z\right| -\left| s\beta z\right| \\= &\,\left| s\beta z^{2}\right| -\left| z\right| +\left| s\beta z\right| -\left| s\beta z\right| \\\ge &\,\left| z\right| (s\beta \left| z\right| -1). \end{aligned}$$

Also for

$$\begin{aligned} z_{1}= &\,(1-\alpha )^{s}z+\alpha ^{s}Q_{c}(u)\\ \left| z_{1}\right|= &\,\left| (1-\alpha )^{s}z+(1-(1-\alpha ))^{s}(u^{2}+c)\right| , \end{aligned}$$

By binomial expansion upto linear terms of $\alpha$ and $(1-\alpha ),$ we obtain

10 $$\begin{aligned} \left| z_{1}\right|= &\,\left| (1-s\alpha )z+(1-s(1-\alpha ))(u^{2}+c)\right| \\= &\,\left| (1-s\alpha )z+(1-s+s\alpha )(u^{2}+c)\right| \\\ge &\,\left| (1-s\alpha )z+s\alpha ((\left| z\right| (s\beta \left| z\right| -1))^{2}+c)\right| \quad\text { (because }1-s+s\alpha \ge s\alpha ) \\ \end{aligned}$$

Since $|z|>2/s\beta$ implies $(s\beta \left| z\right| -1)^{2}>1$ and $\left| z\right| ^{2}(s\beta \left| z\right| -1)^{2}>\left| z\right| ^{2}$ using in (10) we have

11 $$\begin{aligned} \left| z_{1}\right|\ge &\,\left| (1-s\alpha )z+s\alpha (\left| z\right| ^{2}+c)\right| \\\ge &\,\left| s\alpha z^{2}+(1-s\alpha )z\right| -\left| s\alpha c\right| \\\ge &\,\left| s\alpha z^{2}+(1-s\alpha )z\right| -\left| s\alpha z\right| ,\quad\text { because }|z|\ge |c| \\\ge &\,\left| s\alpha z^{2}\right| -\left| (1-s\alpha )z\right| -\left| s\alpha z\right| \\= &\,\left| s\alpha z^{2}\right| -\left| z\right| +\left| s\alpha z\right| -\left| s\alpha z\right| \\\ge &\,\left| z\right| (s\alpha \left| z\right| -1). \end{aligned}$$

Since $|z|>2/s\alpha$ implies $s\alpha \left| z\right| -1>1,$ there exist a number $\lambda >0,$ such that $s\alpha |z|-1>1+\lambda >1.$ Consequently

$$\begin{aligned} \left| z_{1}\right|>&(1+\lambda )\left| z\right| , \\&\vdots \\ \ \left| z_{n}\right|> &\,(1+\lambda )^{n}\left| z\right| . \end{aligned}$$

Hence $\left| z_{n}\right| \longrightarrow \infty$ as $n\rightarrow \infty .$ This completes the proof. $\square$

#### **Corollary 2**

*Suppose that* $|z| \ge |c|,$ $|c|>\frac{2}{s\alpha },|c|>\frac{2}{s\beta }$ *and* $|c|>\frac{ 2}{s\gamma },$ *then, the orbit* $NO_s(Q_c, 0, \alpha , \beta ,\gamma ,s)$ *escapes to infinity*.

Hence the following corollary is the refinement of the escape criterion.

#### **Corollary 3**

(Escape Criterion) *Suppose that* $\left| z\right| >\max \{\left| c\right| ,\frac{2}{s\alpha },\frac{2}{s\beta },\frac{2}{ s\gamma }\},$ *then* $\ \left| z_{n}\right| >(1+\lambda )^{n}\left| z\right|$ *and* $\left| z_{n}\right| \longrightarrow \infty$ *as* $n\rightarrow \infty .$

### Escape criterions for cubic polynomials

We prove the following result for the cubic polynomial $Q_{a,b}(z)=z^{3}+az+b,$ where *a* and *b* are complex numbers, as it is conjugate to all other cubic polynomials.

#### **Theorem 2**

*Suppose* $\left| z\right| \ge \left| b\right|>(\left| a\right| +\frac{2}{s\alpha })^{1/2},\left| z\right| \ge \left| b\right| >(\left| a\right| +\frac{2}{s\beta } )^{1/2}$ *and* $\left| z\right| \ge \left| b\right| >(\left| a\right| +\frac{2}{s\gamma })^{1/2}$ *exist, where* $0<\alpha ,\beta ,\gamma \le 1$ *and* *a*, *b* *are in complex plane. Let* $\ u_{\circ }=u,v_{\circ }=v$ *and* $z_{\circ }=z$ *for* 5 *and* $Q_{a, b}$ *we have* $\left| z_{n}\right| \rightarrow \infty$ *as* *n* $\rightarrow \infty .$

#### *Proof*

Consider

$$\begin{aligned} \left| v\right| =\left| (1-\gamma )^{s}z+\gamma ^{s}Q_{a,b}(z)\right| , \end{aligned}$$

For $$Q_{a,b}(z)=z^{3}+az+b,$$

$$\begin{aligned} \left| v\right|= &\,\left| (1-\gamma )^{s}z+\gamma ^{s}\left( z^{3}+az+b\right) \right| \\= &\,\left| (1-\gamma )^{s}z+(1-(1-\gamma ))^{s}\left( z^{3}+az+b\right) \right| , \end{aligned}$$

By binomial expansion upto linear terms of $\gamma$ and $(1-\gamma )$ we obtain

12 $$\begin{aligned} \left| v\right|\ge &\,\left| (1-s\gamma )z+(1-s(1-\gamma ))\left( z^{3}+az+b\right) \right| \\\ge &\,\left| (1-s\gamma )z+(1-s+s\gamma )\left( z^{3}+az+b\right) \right| \\\ge &\,\left| (1-s\gamma )z+s\gamma \left( z^{3}+az+b\right) \right| ,\quad\text { because }1-s+s\gamma \ge s\gamma \\\ge &\,\left| s\gamma z^{3}+s\gamma az+(1-s\gamma )z\right| -\left| s\gamma b\right| \\\ge &\,\left| s\gamma z^{3}+s\gamma az\right| -\left| (1-s\gamma )z\right| -\left| s\gamma z\right| ,\quad \text {because }\left| z\right| >\left| b\right| \\= &\,\left| s\gamma z^{3}+s\gamma az\right| -\left| z\right| +\left| s\gamma z\right| -\left| s\gamma z\right| \\\ge &\,\ \left| s\gamma z^{3}\right| -\left| s\gamma az\right| -\left| z\right| \\\ge &\,\left| z\right| \left( s\gamma \left( \left| z\right| ^{2}-\left| a\right| \right) -1\right) . \end{aligned}$$

Also for

13 $$\begin{aligned} \left| u\right|= &\,\left| (1-\beta )^{s}z+\beta ^{s}Q_{a,b}(v)\right| \\= &\,\left| (1-\beta )^{s}z+(1-(1-\beta ))^{s}\left( v^{3}+av+b\right) \right| , \end{aligned}$$

By binomial expansion upto linear terms of $\beta$ and $(1-\beta ),$ we obtain

14 $$\begin{aligned} \left| u\right|\ge &\,\left| (1-s\beta )z+(1-s(1-\beta ))\left( v^{3}+av+b\right) \right| \\\ge &\,\left| (1-s\beta )z+(1-s+s\beta )\left( v^{3}+av+b\right) \right| \\\ge &\,\left| (1-s\beta )z+s\beta \left( v^{3}+av+b\right) \right| ,\quad\text { because }1-s+s\beta \ge s\beta \\\ge &\,\left| (1-s\beta )z+s\beta \left( \left( \left| z\right| \left( s\gamma \left( \left| z\right| ^{2}-\left| a\right| \right) -1\right) \right) ^{3}+a\left| z\right| \left( s\gamma \left( \left| z\right| ^{2}-\left| a\right| \right) -1\right) +b\right) \right| , \\ \end{aligned}$$

Since $\left| z\right| >(\left| a\right| +2/s\gamma )^{1/2}$ implies $s\gamma (\left| z\right| ^{2}-\left| a\right| )-1>1,$ so $\left| z\right| (s\gamma (\left| z\right| ^{2}-\left| a\right| )-1)>\left| z\right|$ and $\left| z\right| ^{3}(\gamma (\left| z\right| ^{2}-\left| a\right| )-1)^{3}>\left| z\right| ^{3}$ using in (14) we have

15 $$\begin{aligned} \left| u\right|\ge &\,\left| (1-s\beta )z+s\beta \left( \left| z\right| ^{3}+a\left| z\right| +b\right) \right| \\\ge &\,\left| s\beta \left| z\right| ^{3}+s\beta a\left| z\right| +(1-s\beta )z\right| -\left| s\beta b\right| \\\ge &\,\left| s\beta \left| z\right| ^{3}+s\beta a\left| z\right| +(1-s\beta )z\right| -\left| s\beta z\right| ,\quad\text { because }\left| z\right| >\left| b\right| \\\ge &\,\left| s\beta \left| z\right| ^{3}\right| -\left| s\beta a\left| z\right| \right| -\left| (1-s\beta )z\right| -\left| s\beta z\right| \\\ge &\,\ \left| s\beta \left| z\right| ^{3}\right| -\left| s\beta a\left| z\right| \right| -\left| z\right| +\left| s\beta z\right| -\left| s\beta z\right| \\\ge &\,\left| z\right| \left( s\beta \left( \left| z\right| ^{2}-\left| a\right| \right) -1\right) . \end{aligned}$$

Now,

$$\begin{aligned} z_{1}= &\,(1-\alpha )^{s}z+\alpha ^{s}Q_{a,b}(u) \\ \left| z_{1}\right|= &\,\left| (1-\alpha )^{s}z+(1-(1-\alpha ))^{s}\left( u^{3}+au+b\right) \right| , \end{aligned}$$

By binomial expansion upto linear terms of $\alpha$ and $(1-\alpha ),$ we obtain

16 $$\begin{aligned} \left| z_{1}\right|= &\,\left| (1-s\alpha )z+(1-s(1-\alpha ))\left( u^{3}+au+b\right) \right| \\= &\,\left| (1-s\alpha )z+(1-s+s\alpha )\left( u^{3}+au+b\right) \right| \\\ge &\,\left| (1-s\alpha )z+s\alpha \left( u^{3}+au+b\right) \right| ,\quad\text { because }1-s+s\alpha \ge s\alpha \\\ge &\,\left| (1-s\alpha )z+s\alpha \left( \left( \left| z\right| \left( s\beta \left( \left| z\right| ^{2}-\left| a\right| \right) -1\right) \right) ^{3}+a\left| z\right| \left( s\beta \left( \left| z\right| ^{2}-\left| a\right| \right) -1\right) +b\right) \right| , \\&\end{aligned}$$

Since $\left| z\right| >(\left| a\right| +2/s\beta )^{1/2}$ implies $s\beta (\left| z\right| ^{2}-\left| a\right| )-1>1,$ so $\left| z\right| (s\beta (\left| z\right| ^{2}-\left| a\right| )-1)>\left| z\right|$ and $\left| z\right| ^{3}(s\beta (\left| z\right| ^{2}-\left| a\right| )-1)>\left| z\right| ^{3}$ using in (16) we have

17 $$\begin{aligned} \left| z_{1}\right|\ge &\,\left| (1-s\alpha )z+s\alpha \left( \left| z\right| ^{3}+a\left| z\right| +b\right) \right| \\\ge &\,\left| s\alpha \left| z\right| ^{3}+s\alpha a\left| z\right| +(1-s\alpha )z\right| -\left| s\alpha b\right| \\\ge &\,\left| s\alpha \left| z\right| ^{3}+s\alpha a\left| z\right| +(1-s\alpha )z\right| -\left| s\alpha z\right| , \quad\text { because }\left| z\right| >\left| b\right| \\\ge &\,\left| s\alpha \left| z\right| ^{3}\right| -\left| s\alpha a\left| z\right| \right| -\left| (1-s\alpha )z\right| -\left| s\alpha z\right| \\\ge &\,\ \left| s\alpha \left| z\right| ^{3}\right| -\left| s\alpha a\left| z\right| \right| -\left| z\right| +\left| s\alpha z\right| -\left| s\alpha z\right| \\\ge &\,\left| z\right| \left( s\alpha \left( \left| z\right| ^{2}-\left| a\right| \right) -1\right) . \end{aligned}$$

Since $\left| z\right| >(\left| a\right| +2/s\alpha )^{1/2}$ implies $s\alpha (\left| z\right| ^{2}-\left| a\right| )-1>1.$ Hence there exists $\lambda >1$ such that, $\left| z_{1}\right| >\lambda \left| z\right| .$ Repeating the argument *n* times, we get $\left| z_{n}\right| >\lambda ^{n}\left| z\right|$. Therefore, the orbit of *z* under the cubic polynomial $Q_{a,b}(z),$ tends to infinity. This completes the proof. $\square$

#### **Corollary 4**

(Escape criterion) *Let* $Q_{a,b}(z)=z^{3}+az+b,$ *where* *a* *and* *b* *are complex numbers*. *Suppose* $\left| z\right| >\max \left\{ \left| b\right| ,\left( \left| a\right| +\frac{2}{s\alpha }\right) ^{1/2},\left( \left| a\right| +\frac{2}{s\beta }\right) ^{1/2},\left( \left| a\right| +\frac{2}{s\gamma }\right) ^{1/2}\right\}$ then $\left| z_{n}\right| \rightarrow \infty$ as $n\rightarrow \infty .$

### A general escape criterion

We will obtain a general escape criterion for polynomials of the form $G_{c}(z)=z^{k+1}+c.$

#### **Theorem 3**

*For general function* $G_{c}(z)=z^{k+1}+c,k=1,2,3,\ldots ,$ *suppose that* $|z|\ge |c|>\left( \frac{2}{s\alpha }\right) ^{1/k}$, $|z|\ge |c|>\left( \frac{2}{s\beta }\right) ^{1/k}$ *and* $|z|\ge |c|>\left( \frac{2}{s\gamma }\right) ^{1/k}$ *where* *c* *be a complex number and* $0<\alpha ,\beta ,\gamma \le 1$. *Let* $\ u_{\circ }=u,v_{\circ }=v$ *and* $z_{\circ }=z$ *then from the iteration* 5, *we have* $\left| z_{n}\right| \rightarrow \infty$ *as* *n* $\rightarrow \infty .$

#### *Proof*

Let $G_{c}(z)=z^{k+1}+c$ and $\left| z\right| \ge \left| c\right|>\left( \frac{2}{s\alpha }\right) ^{1/k},\left| z\right| \ge \left| c\right| >\left( \frac{2}{s\beta }\right) ^{1/k}$ as well as $\left| z\right| \ge \left| c\right| >\left( \frac{2}{s\gamma }\right) ^{1/k}$ exists then for $G_{c}(z)=z^{k+1}+c,$ consider

$$\begin{aligned} \left| v\right|= &\,\left| (1-\gamma )^{s}z+\gamma ^{s}\left( z^{k+1}+c\right) \right| \\= &\,\left| (1-\gamma )^{s}z+(1-(1-\gamma ))^{s}\left( z^{k+1}+c\right) \right| , \end{aligned}$$

By binomial expansion upto linear terms of $\gamma$ and $(1-\gamma ),$ we obtain

18 $$\begin{aligned} \left| v\right|\ge &\,\left| (1-s\gamma )z+(1-s(1-\gamma ))\left( z^{k+1}+c\right) \right| \\\ge &\,\left| (1-s\gamma )z+(1-s+s\gamma )\left( z^{k+1}+c\right) \right| \\\ge &\,\left| (1-s\gamma )z+s\gamma \left( z^{k+1}+c\right) \right| ,\quad\text { because }1-s+s\gamma \ge s\gamma \\\ge &\,\left| s\gamma z^{k+1}+(1-s\gamma )z\right| -\left| s\gamma c\right| \\\ge &\,\left| s\gamma z^{k+1}+(1-s\gamma )z\right| -\left| s\gamma z\right| ,\quad\text { because }|z|\ge |c| \\\ge &\,\left| s\gamma z^{k+1}\right| -\left| (1-s\gamma )z\right| -\left| s\gamma z\right| \\= &\,\left| s\gamma z^{k+1}\right| -\left| z\right| +\left| s\gamma z\right| -\left| s\gamma z\right| \\\ge &\,\left| z\right| \left( s\gamma \left| z\right| ^{k}-1\right) . \end{aligned}$$

and

19 $$\begin{aligned} \left| u\right|= &\,\left| (1-\beta )^{s}z+\beta ^{s}G_{c}(v)\right| \\= &\,\left| (1-\beta )^{s}z+(1-(1-\beta ))^{s}\left( v^{k+1}+c\right) \right| , \end{aligned}$$

By binomial expansion upto linear terms of $\beta$ and $(1-\beta ),$ we obtain

20 $$\begin{aligned} \left| u\right|\ge &\,\left| (1-s\beta )z+(1-s(1-\beta ))\left( v^{k+1}+c\right) \right| \\\ge &\,\left| (1-s\beta )z+(1-s+s\beta )\left( v^{k+1}+c\right) \right| \\\ge &\,\left| (1-s\beta )z+s\beta \left( \left( \left| z\right| \left( s\gamma \left| z\right| ^{k}-1\right) \right) ^{k+1}+c\right) \right| ,\quad\text { because }1-s+s\beta \ge s\beta \\ \end{aligned}$$

Since $|z|>(2/s\gamma )^{1/k}$ implies $s\gamma \left| z\right| ^{k}-1>1$ also ($s\gamma \left| z\right| ^{k}-1)^{2}>1$ and $\left| z\right| ^{k+1}(s\gamma \left| z\right| -1)^{2}>$ $\left| z\right| ^{k+1}$ using this in (20) we have

21 $$\begin{aligned} \left| u\right|\ge &\,\left| (1-s\beta )z+s\beta \left( \left| z\right| ^{k+1}+c\right) \right| \\\ge &\,\left| s\beta z^{k+1}+(1-s\beta )z\right| -\left| s\beta c\right| \\\ge &\,\left| s\beta z^{k+1}+(1-s\beta )z\right| -\left| s\beta z\right| ,\quad\text { because }|z|\ge |c| \\\ge &\,\left| s\beta z^{k+1}\right| -\left| (1-s\beta )z\right| -\left| s\beta z\right| \\= &\,\left| s\beta z^{k+1}\right| -\left| z\right| +\left| s\beta z\right| -\left| s\beta z\right| \\\ge &\,\left| z\right| \left( s\beta \left| z\right| ^{k}-1\right) . \end{aligned}$$

Also for

$$\begin{aligned} z_{1}= &\,(1-\alpha )^{s}z+\alpha ^{s}G_{c}(u) \\ \left| z_{1}\right|= &\,\left| (1-\alpha )^{s}z+(1-(1-\alpha ))^{s}\left( u^{k+1}+c\right) \right| , \end{aligned}$$

By binomial expansion upto linear terms of $\alpha$ and $(1-\alpha ),$ we obtain

22 $$\begin{aligned} \left| z_{1}\right|\ge &\,\left| (1-s\alpha )z+(1-s(1-\alpha ))\left( u^{k+1}+c\right) \right| \\\ge &\,\left| (1-s\alpha )z+(1-s+s\alpha )\left( u^{k+1}+c\right) \right| \\\ge &\,\left| (1-s\alpha )z+s\alpha \left( \left( \left| z\right| \left( \beta \left| z\right| -1\right) \right) ^{k+1}+c\right) \right| ,\quad\text { because } 1-s+s\alpha \ge s\alpha \\&\end{aligned}$$

Since $|z|>(2/s\beta )^{1/k}$ implies $(s\beta \left| z\right| ^{k}-1)^{k+1}>1$ and $\left| z\right| ^{k+1}(s\beta \left| z\right| ^{k}-1)^{k+1}>\left| z\right| ^{k+1}$ using in (22) we have

23 $$\begin{aligned} \left| z_{1}\right|\ge &\,\left| (1-s\alpha )z+s\alpha \left( \left| z\right| ^{k+1}+c\right) \right| \\\ge &\,\left| s\alpha z^{k+1}+(1-s\alpha )z\right| -\left| s\alpha c\right| \\\ge &\,\left| s\alpha z^{k+1}+(1-s\alpha )z\right| -\left| s\alpha z\right|,\quad \text { because }|z|\ge |c| \\\ge &\,\left| s\alpha z^{k+1}\right| -\left| (1-s\alpha )z\right| -\left| s\alpha z\right| \\= &\,\left| s\alpha z^{k+1}\right| -\left| z\right| +\left| s\alpha z\right| -\left| s\alpha z\right| \\\ge &\,\left| z\right| \left( s\alpha \left| z\right| ^{k}-1\right) . \end{aligned}$$

Since $|z|>(2/s\alpha )^{1/k}$ implies $s\alpha \left| z\right| ^{k}-1>1,$ there exist a number $\lambda >0,$ such that $s\alpha |z|^{k}-1>1+\lambda >1.$ Consequently

$$\begin{aligned} \left| z_{1}\right|>&(1+\lambda )\left| z\right| , \\&\vdots \\ \ \left| z_{n}\right|> &\,(1+\lambda )^{n}\left| z\right| . \end{aligned}$$

Hence $\left| z_{n}\right| \longrightarrow \infty$ as $n\rightarrow \infty$. This completes the proof. $\square$

#### **Corollary 5**

*Suppose that* $|c|>\left( \frac{2}{s\alpha }\right) ^{1/k},|c|>\left( \frac{2}{s\beta }\right) ^{1/k}$ *and* $|c|>\left( \frac{2}{s\gamma }\right) ^{1/k}$ *exists, then the orbit* $NO_s(G_{c},0,\alpha ,\beta ,\gamma ,s )$ *escape to infinity*.

This corollary gives an algorithm for computing the Julia sets and Mandelbrot sets for the functions of the form $G_{c}(z)=z^{k+1}+c,k=1,2,3,\ldots$

## Generation of Julia sets and Mandelbrot sets

In this section we present some Mandelbrot sets for quadratic and cubic functions by using the computational work in Mathematica 9.0. and following code

### Mandelbrot sets for the quadratic polynomial $Q_{c}(z)=z^{2}+c$

**Fig. 1:**
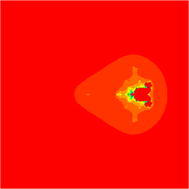
Quadratic Mandelbrot set for $\alpha = \beta = \gamma =0.3$ and $s=0.1$

**Fig. 2:**
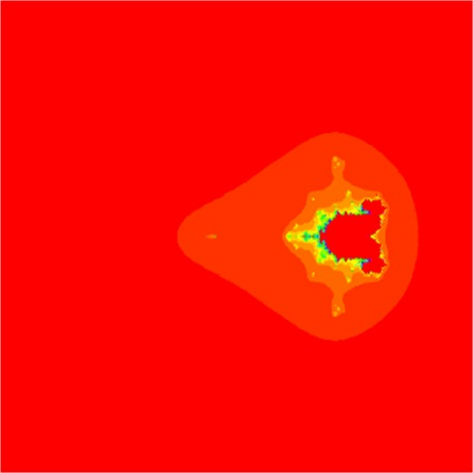
Quadratic Mandelbrot set for $\alpha = \beta = \gamma =0.3$ and $s=0.2$

**Fig. 3:**
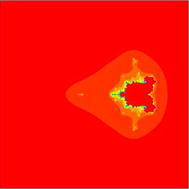
Quadratic Mandelbrot set for $\alpha = \beta = \gamma =0.3$ and $s=0.3$

**Fig. 4:**
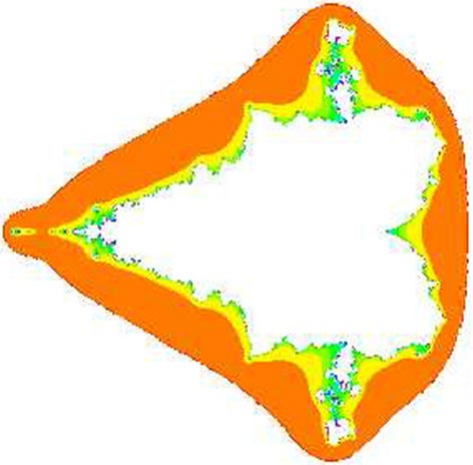
Quadratic Mandelbrot set for $\alpha = \beta = \gamma =0.3$ and $s=0.8$

**Fig. 5:**
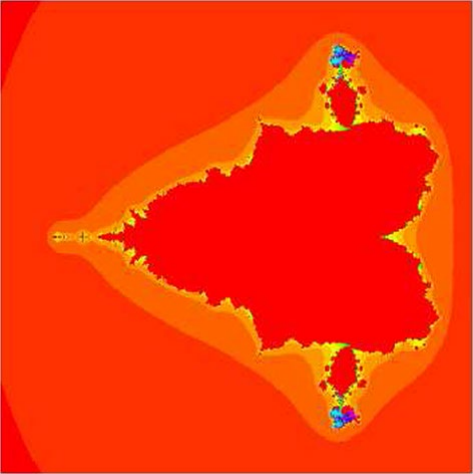
Quadratic Mandelbrot set for$\alpha = \beta = \gamma =0.3$ and $s=0.9$

**Fig. 6:**
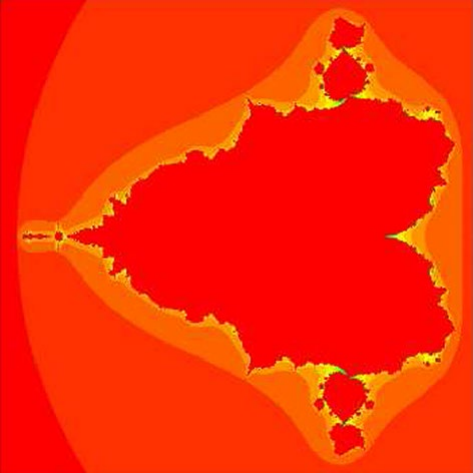
Quadratic Mandelbrot set for $\alpha = \beta = \gamma =0.3$ and $s=1$

In Figs. 1, 2, 3, 4, 5, and 6, quadratic Mandelbrot sets are presented in Noor orbit with s-convexity by using maximum number of iterations 30 and grid $[-7,2]\times [-4,4]$.

### Mandelbrot sets for the cubic polynomial $Q_{0,c}(z)=z^{3}+c$

In Figs. 7, 8, 9, 10, 11, 12, 13, and 14 cubic Mandelbrot sets are presented in Noor orbit with s-convexity by using maximum number of iterations 30 and grid $[-3.5,3.5]\times [ -6,6]$.

**Fig. 7:**
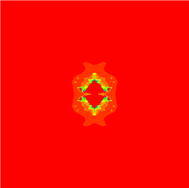
Cubic Mandelbrot set for $\alpha =\beta =\gamma =0.05$ and $s=0.2$

**Fig. 8:**
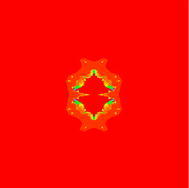
Cubic Mandelbrot set for $\alpha =\beta =\gamma =0.05$ and $s=0.3$

**Fig. 9:**
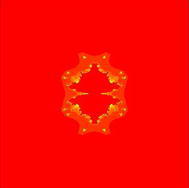
Cubic Mandelbrot set for $\alpha =\beta =\gamma =0.05$ and $s=0.4$

**Fig. 10:**
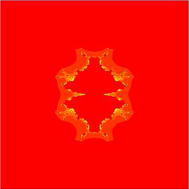
Cubic Mandelbrot set for $\alpha =\beta =\gamma =0.05$ and $s=0.5$

**Fig. 11:**
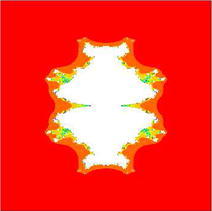
Cubic Mandelbrot set for $\alpha =\beta =\gamma =0.05$ and $s=0.7$

**Fig. 12:**
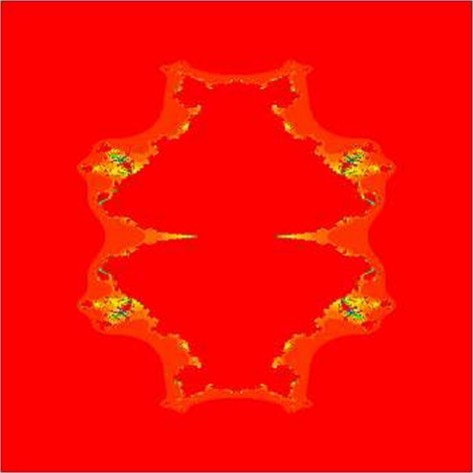
Cubic Mandelbrot set for $\alpha =\beta =\gamma =0.05$ and $s=0.8$

**Fig. 13:**
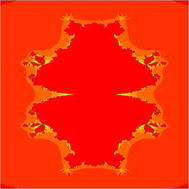
Cubic Mandelbrot set for $\alpha =\beta =\gamma =0.05$ and $s=0.9$

**Fig. 14:**
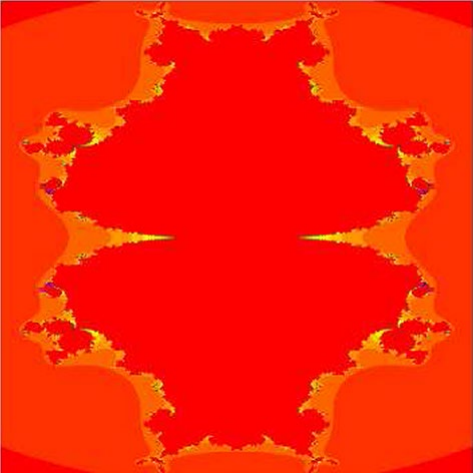
Cubic Mandelbrot set for $\alpha =\beta =\gamma =0.05$ and $s=1$

### Julia sets for the quadratic polynomial $Q_{c}(z)=z^{2}+c$

**Fig. 15:**
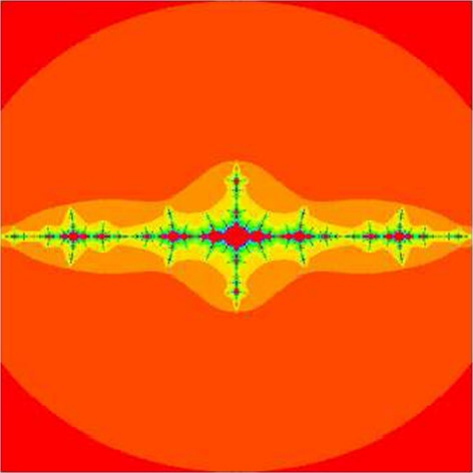
Quadratic Julia set for $\alpha =1, \beta =1, \gamma =1$ and $c=-1.38$

**Fig. 16:**
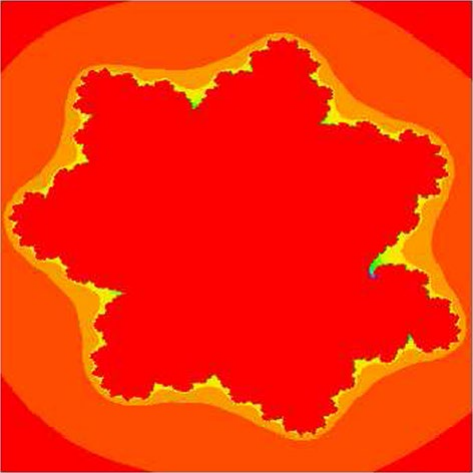
Quadratic Julia set for $\alpha =0.1, \beta =0.9, \gamma =0.9$ and $c=0.23+0.23i$

**Fig. 17:**
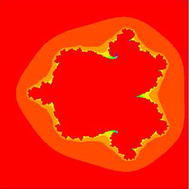
Quadratic Julia set for $\alpha =0.1, \beta =0.1, \gamma =0.9$ and $c=-0.23+0.23i$

**Fig. 18:**
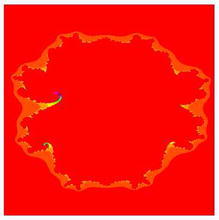
Quadratic Julia set for $\alpha =0.1, \beta =0.1, \gamma =0.5$ and $c=-0.5-0.5i$

Quadratic Julia sets are presented in Figs. 15, 16, 17, and 18 for Noor iteration scheme with s-convexity by using maximum number of iterations 20 and $s=1$.

## Conclusions

In this paper we presented new fixed point results for Noor iteration with s-convexity in the generation of fractals (Julia sets and Mandelbrot sets). The new escape criterions have been established for complex quadratic, cubic, and ($k+1$)th degree polynomials. Very interesting changes in Mandelbrot sets can be seen when s varies from lowest to higher values but variation of s does not effect Julia sets. The results of escape criterions for Julia sets and Mandelbrot sets in Noor orbit presented in Ashish and Chugh (2014) are as special case of our results for $s=1$.
